# Mechanical circulatory support for shock: A little bit better is just not enough!

**DOI:** 10.1007/s12471-020-01411-3

**Published:** 2020-03-18

**Authors:** M. Karami, J. P. S. Henriques

**Affiliations:** Heart Center, Department of Cardiology, Amsterdam Cardiovascular Sciences, Amsterdam UMC, University of Amsterdam, Amsterdam, The Netherlands

Cardiogenic shock remains the leading cause of death in patients admitted for acute myocardial infarction [1]. In the past two decades, the mortality has remained unchanged at around 40–50%. Apart from early revascularisation, there is no evidence that the current treatment strategies are effective in improving survival [1].

Cardiogenic shock is a clinical state of critical end-organ hypoperfusion and hypoxia, caused by a decreased cardiac output due to myocardial dysfunction. As such, therapy for cardiogenic shock aims to increase cardiac output and blood pressure. Fluids, vasopressors and inotropes are used as a first-line treatment strategy for haemodynamic optimisation. However, the use of vasopressors and inotropes in patients with acute coronary disease is not ideal, as these drugs increase the myocardial oxygen demand which could potentially worsen the cardiac function. Moreover, in experimental studies, the use of vasopressors and inotropes was associated with myocardial infarct size expansion and increased risk of organ failure. Mechanical support devices are frequently used as an alternative treatment strategy to enable stable haemodynamics and reduce the need for these drugs.

The goals of mechanical support in cardiogenic shock are maintaining sufficient perfusion of myocardium and other end-organs, preventing or reducing multi-organ failure and improving survival. Although an increasing number of support devices has become available over the years, randomised data on efficacy remains limited. The intra-aortic balloon pump (IABP) is ineffective in the setting of cardiogenic shock and only offers modest haemodynamic support. Although the Impella devices are able to deliver higher output than the IABP, this beneficial effect has not translated into better clinical outcome either. A recent meta-analysis found no evidence for improved survival with the Impella and Tandemheart devices (*n* = 77) compared with the IABP (*n* = 71) in patients with cardiogenic shock [2].

In this issue, van Dort et al. performed an extensive review of the current evidence on haemodynamic efficacy of Impella support in patients with cardiogenic shock [3]. In their meta-analysis, they confirmed previous findings that the Impella had a beneficial effect on haemodynamic parameters (i.e. cardiac power [CP] and cardiac power index). They also confirmed that, although Impella yielded better haemodynamics, this beneficial effect did not translate into an improved survival.

A number of reasons could explain the lack of evidence on improved outcomes in patients supported by Impella or other mechanical circulatory support devices. First, does the amount of haemodynamic support provided by the device suffice? The Impella is capable of yielding better circulatory support than the IABP, but the clinical relevance of the reported haemodynamic improvement by Impella in the current and previous papers is not clear. Previously, we found there was a trend toward better clinical outcome in patients treated with larger Impella devices (6-month survival rates: Impella 2.5 30%, Impella CP 38.5% and Impella 5.0 60%) [4]. Possibly, a support device that offers an even higher output, such as the extracorporeal membrane oxygenation (ECMO) system, would achieve a greater improvement in haemodynamics and improve outcome. Nonetheless, there is no evidence that clinical outcome with ECMO is superior to outcome with Impella, and ECMO is actually associated with a higher rate of complications [5].

Second, are we selecting the right patients? The condition of patients included in this study could have been too severe to actually derive a benefit from mechanical support. Frequently, patients in cardiogenic shock suffer from cardiac arrest with irreversible neurological damage. Or in some cases, severe sepsis with multi-organ failure may develop, which cannot be ameliorated by providing mechanical support. Furthermore, some patients only have a mild form of cardiogenic shock and will not benefit from circulatory support, but will be at risk for severe device-related complications. It has been speculated that approximately 20% of cardiogenic shock patients could benefit from a mechanical support device [1]. To show an efficacy of support, therefore, selecting the appropriate patients and the timing of initiation are key. Preferably, mechanical support should be initiated before full haemometabolic cardiogenic shock develops. Unfortunately, there are no early and reliable clinical or biological markers that enable adequate patient selection.

Third, what is the effect of pharmacological therapy? It is unclear whether the use of vasopressors and inotropes leads to better outcomes in cardiogenic shock, but adverse effect of these drugs are frequently reported. These routinely used drugs need to be carefully evaluated as they may also mitigate effects from other interventions.

Fourth, “primum non nocere”. We are not able to show better clinical outcome with the use of mechanical support devices, however, deployment of this type of therapy is associated with complications. Device-related complications such as haemolysis and bleeding may occur, which can lead to an increased morbidity and mortality. In a recent comprehensive study that used real-world data (JAMA, 2020), the introduction of mechanical support did not result in improved survival, but was actually associated with a higher risk of major bleeding and in-hospital mortality [6].

Cardiogenic shock is a condition of low cardiac output. Impella and other mechanical support devices have shown to improve cardiac output, but thus far none of the devices has shown better clinical outcome. Nevertheless, we believe that the routine use of quick and powerful mechanical support devices in dedicated cardiogenic shock centres will improve clinical outcome, but we still have a long way to go. We must objectively evaluate current treatment strategies and be willing to abandon our old beliefs, as previously with the use of IABP in cardiogenic shock. It is necessary to perform clinical trials in cardiogenic shock in order to move away from the current impasse. Adequate patient selection, early initiation of support, probably a much higher level of support, low risk of complications and evaluation of treatment strategies (pharmacological and mechanical) in proper randomised trials with relevant clinical end-points are important. A little bit of improvement in haemodynamics is just not enough to improve outcomes of patients in cardiogenic shock.
